# Anchoring durum wheat diversity in the reality of traditional agricultural systems: varieties, seed management, and farmers’ perception in two Moroccan regions

**DOI:** 10.1186/1746-4269-10-58

**Published:** 2014-07-15

**Authors:** Lamyae Chentoufi, Ali Sahri, Mustapha Arbaoui, Loubna Belqadi, Ahmed Birouk, Pierre Roumet, Marie-Hélène Muller

**Affiliations:** 1 Département de Production, Protection et Biotechnologies Végétales, Institut Agronomique et Vétérinaire Hassan II, B.P. 6202, Rabat-Instituts, Rabat, Morocco; 2 INRA, UMR 1334, Amélioration Génétique et Adaptation des Plantes méditerranéennes et tropicales (AGAP), 2 place Pierre Viala, F-34060 Montpellier Cedex 1, France

**Keywords:** Traditional agriculture, Triticum turgidum, Varietal diversity, *In situ* conservation, Interviews, Farmers’ knowledge

## Abstract

**Background:**

Traditional agrosystems are the places were crop species have evolved and continue to evolve under a combination of human and environmental pressures. A better knowledge of the mechanisms underlying the dynamics of crop diversity in these agrosystems is crucial to sustain food security and farmers’ self-reliance. It requires as a first step, anchoring a description of the available diversity in its geographical, environmental, cultural and socio-economic context.

**Methods:**

We conducted interviews with farmers cultivating durum wheat in two contrasted traditional agrosystems of Morocco in the Pre-Rif (163 farmers) and in the oases of the Atlas Mountains (110 farmers). We documented the varietal diversity of durum wheat, the main characteristics of the farms, the farming and seed management practices applied to durum wheat, and the farmers’ perception of their varieties.

**Results:**

As expected in traditional agrosystems, farmers largely practiced diversified subsistence agriculture on small plots and relied on on-farm seed production or informal seed exchange networks. Heterogeneity nevertheless prevailed on many variables, especially on the modernization of practices in the Pre-Rif region. Fourteen (resp. 11) traditional and 5 (resp. 3) modern varieties were identified in the Pre-Rif region (resp. in the Atlas Mountains). The majority of farmers grew a single variety, and most traditional varieties were distributed in restricted geographical areas. At the farm level, more than half of the varieties were renewed in the last decade in the Pre-Rif, a more rapid renewal than in the Atlas Mountain. Modern varieties were more prevalent in the Pre-Rif region and were integrated in the traditional practices of seed production, selection and exchange. They were clearly distinguished by the farmers from the landraces, the last ones being appreciated for their quality traits.

**Conclusions:**

The surveyed traditional agrosystems constitute open, dynamic and heterogeneous entities. We suggest that competing factors could favour or limit the cultivation of improved varieties and the erosion of original durum wheat diversity. This first description opens the way to focused further investigations, including complementing variety names with cultural, genetic and phenotypic information and unravelling the multidimensional factors and consequences of modern variety adoption.

## Background

It is now widely recognized that the conservation of agrobiodiversity is of major importance for the future of agriculture and food security. In the last decades, this has stimulated the development of numerous collections, research centers and gene banks [1,2]. But agrobiodiversity is not just a quantity but is essentially a set of processes that govern the permanent evolution, regeneration and losses of crop species, of varieties, of alleles. In traditional agrosystems, smallholder farmers still grow, use and produce their own seed, and cultivate a diversity of crop species with few inputs from outside (synthetic fertilizers, improved seeds, capital or scientific knowledge) fertilizers, improved seeds, capital or scientific knowledge, [3,4]. The farmers’ varieties are denoted as traditional varieties, or landraces. Since the beginning of agriculture, these agrosystems have been the key place where crop species have evolved in interaction with environmental factors and human practices [5,6]. Traditional agrosystems have almost disappeared in developed countries, where modern varieties, resulting from formal crop breeding programs, gradually replaced the landraces but see [7]. They are however still important elsewhere, especially in marginal areas where modern breeding has not yet provided adapted varieties. They provide food for a vast quantity of people and are a reservoir of crop diversity and also of practices and evolutionary processes [3].

In a context of global environmental and social change, it is important to understand what is actually going on in traditional agrosystems, namely the processes at work and the way they are affected and threatened. Such understanding is crucial to be able to accompany the practices towards sustainable food production [8], as well as to inspire the design of more sustainable systems in developed countries as well [3,9]. For such purposes, studies have to go beyond the description of diversity, whatever the level of investigation (species and varieties names, phenotypic traits or molecular markers) and envision the context in which it has been produced, that is the farmers’ work and knowledge [10].

In accordance with this objective, studies have been conducted in the last years, involving farmer-centered interviews that associated the inventory of cultivated varieties with information on the farmers, its farm and its agroecological and social environment. Jarvis et al. [11] produced and compiled such data for 27 crop species worldwide and estimated indicators classically used for biodiversity assessment (e.g. richness, evenness, divergence) for crop varieties at the farm and community levels. This provided a first worldwide gauge of crop-varietal diversity on farm, including the diversity of landraces and the occurrence and importance of the introduction of modern varieties. Their study drew the framework for future comparisons with other crops and species e.g. using the same indicators, [12].

Simultaneously fine-scale studies were able to investigate more precisely the impact of the physical and socio-economic settings on the level and distribution of diversity, for instance the relationships between the farmers’ wealth and the number of landraces they grow [13], or the factors of adoption of modern varieties [14]. Finally, studies of the farmers’ perception of varietal names and of the way they actually manage their seed lots [15,16] have shown that the reality behind varietal names is not always the same depending on the species and the country [11]. Thus, even if investigating the diversity of varietal names is a convenient and necessary starting point, it will need to be complemented with genetic and phenotypic characterization [16,17].

The present study focused on durum wheat (*Triticum turgidum* ssp. *durum*) cultivated in traditional agrosystems of two regions of Morocco. Durum wheat is a subspecies of tetraploid wheat (*Triticum turgidum*), a selfing species domesticated around the 8^th^ millennium BC in the Fertile Crescent [18]. In North Africa, cereal systems based on durum wheat may date from the first dissemination of the species with the early agricultural movements (from 5000 BC). Durum wheat farming in North Africa became a reference for Romans since it was considered as a “bread basket of Rome” Raven 1993 cited by [19]. Since this period, durum wheat has been an important crop (about 1 million ha/year, 20% of cereals area in Morocco, http://www.agriculture.gov.ma) and a major source of staple food in Maghreb (traditional bread, couscous, pasta). In the seventies, the green revolution impacted cereal cultivation in the plains, with modern varieties developed by national or international centers (ICARDA, CIMMYT). But traditional agriculture persisted in marginal regions such as the Atlas and Rif mountains. Wide scale molecular diversity analyses have recently revealed that variability existed among Moroccan landraces, and that this variability was partly associated with geography and environment [20,21]. However, the studied accessions were not anchored within the agro-ecosystems they came from, which hindered the potentiality to fully characterize the actual diversity present in these agrosystems, as well as the processes at work.

We chose to focus on two contrasted regions: the Pre-Rif region is a contact zone between traditional and modern agriculture, where modern varieties are being introduced and promoted by the Agricultural Extension Offices. The oases of the Atlas Mountains correspond to a more isolated area, and have been kept farther from the products of modern breeding. We conducted interviews with farmers to collect information on the diversity of practices and social context, on the diversity of cultivated varieties, and on the way farmers appreciate their qualities. Our objectives were to document varietal diversity and its spatial variation, as initiated by Jarvis et al. [11], and to link this diversity with the agronomic and socio-economic context. We also aimed to open the way to pertinent questions on how these agrosystems function, on the main factors acting on them, and on the link between farmers’ management of their seed and the local diversity.

## Methods

### The study areas

The first study region is a part of the Pre-Rif region of Northern Morocco. It is characterized by a mountainous topography and a semi-arid climate. Precipitation varies between 400 and 600 mm a year. The dominant soils are calcareous shales and associated lithosols regosols [22]. The region is dominated by cereals, combined with arboriculture (olive, fig and almond trees) and livestock (cattle, sheep and goats). Agriculture is mainly rain-fed. Irrigation does not exceed 4% of the agricultural area of the region [23]. The surveyed area is at the interface between the plains where modern agriculture is increasing in importance and the Rif Mountains were traditional agriculture and marginal conditions prevail.

The second study region (oases of the Atlas Mountains) is located in the eastern High Atlas (Midelt province) along Imilchil valley. This valley is characterized by a very pronounced aridity and a continental climate with large temperature variations [24]. Annual precipitation ranges from 100 to 400 mm. The dominant soils are lithosols regosols in combination with brown soil and sierozems [22]. Agriculture in the region is irrigated. It is based on cereals and fodder crops [25]. Before the building of a road (2008–2009), this valley remained relatively isolated from the others regions.

**Figure 1:**
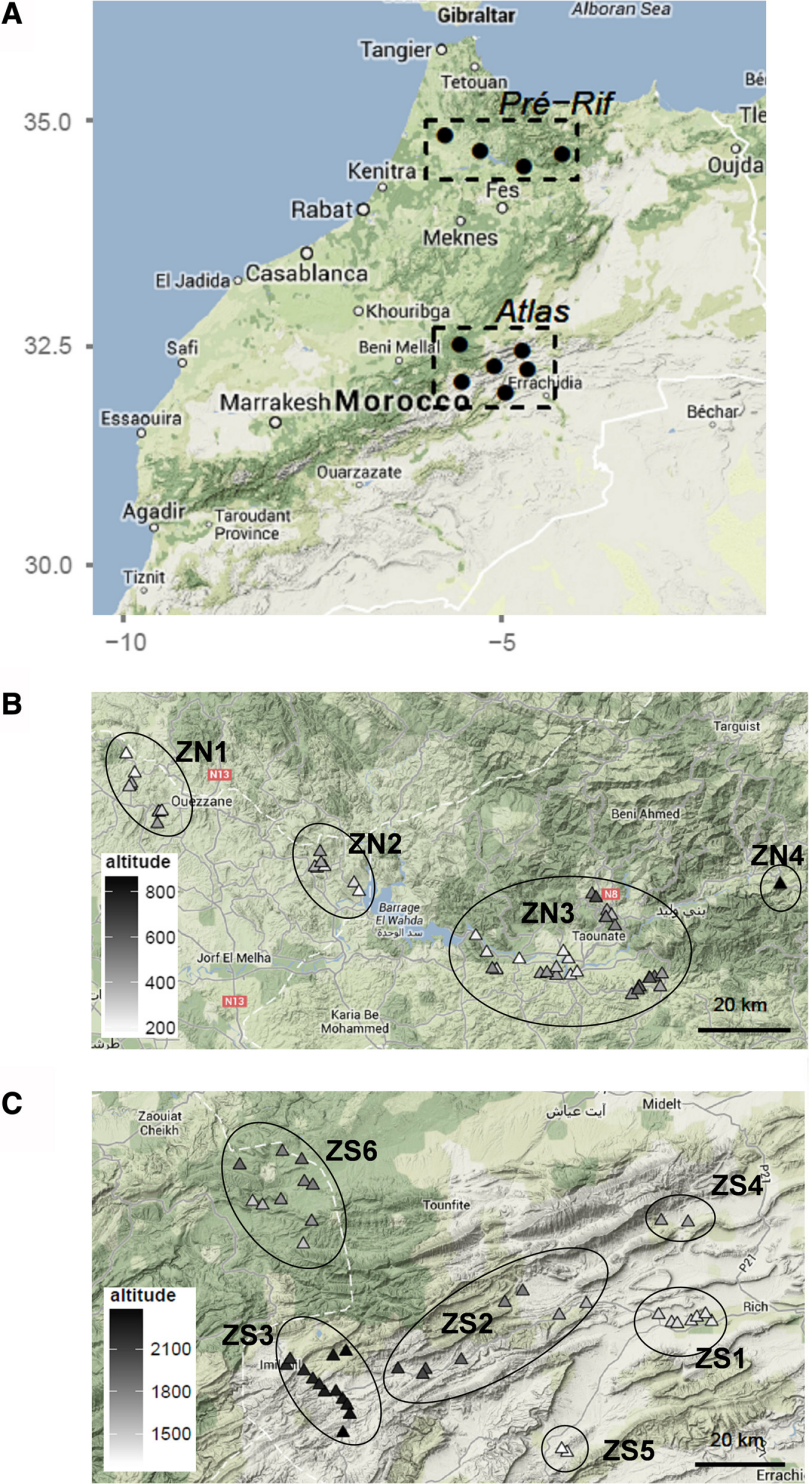
**Study areas.** Map of Northern Morocco showing the locations of the two study regions **(A)**. Details of each region showing the zones and the surveyed villages (**B**. Pre-Rif, **C**. Atlas Mountains). Symbols are colored in accordance with the villages’ altitude. The map backgrounds have been extracted from Google Map.

In the Pre-Rif, we conducted reconnaissance tours with technicians of the agricultural extension office, in order to identify and sample villages where farmers still use traditional durum wheat varieties. Four zones were defined by grouping geographically close villages. ZN1 and ZN2 are located in the province of Ouazzane, ZN3 and ZN4 are located in Taounate (Figure 1). The population of the area is Arab and comprises two ethnical groups, Jbala (all zones) and Hyanya (ZN3). These groups are not isolated from each other: economic exchanges as well as weddings are common between them (Technical Center Taounate, pers. Comm.) [26].

In the Atlas Mountains, we first chose to focus on the Ziz valley, which had been investigated in a previous study [27]: Zones ZS1, ZS2 and ZS3 (Er-Rich, Amouguer-Outerbate, Imilchil) are located along an altitudinal gradient in this valley. Three additional zones were selected following a reconnaissance tour: ZS4 and ZS5 (Zaouiat Sidi Hamza Amellagou North and South-West of Er-Rich) were signaled as containing diverse traditional varieties, potentially original compared to three initial zones; ZS6 (Aghbala) was identified as a source of supply of traditional durum wheat seed for the region of Imilchil (ZS3) (Figure 1). Villages were randomly selected within these zones, since all farmers grow traditional varieties (Table 1). The population of this area is Berber and represented historically by 5 major tribes sharing Tamazight language and partially overlapping geographically: Ait Izdeg (ZS1, ZS2 and ZS4); Ait Merrhad (ZS1, ZS4, ZS5) Ait Yahia (ZS2, ZS6) Ait Haddidou (ZS3) and Ait Sokhman (ZS6) [28].

**Table 1:** Summary of sample design

**Region**	**Zone**	**Number of visited villages**	**Number of interviewed farmers**	**Altitude range (m)**
**Pre-Rif**	**ZN1**	9	51	187-428
	**ZN2**	7	10	191-422
	**ZN3**	30	86	180-650
	**ZN4**	1	16	872
**Total Pre-Rif**		47	163	
**Atlas Mountains**	**ZS1**	7	15	1385-1464
	**ZS2**	8	22	1593-2133
	**ZS3**	13	27	2160-2387
	**ZS4**	2	8	1662-1674
	**ZS5**	2	6	1293-1296
	**ZS6**	10	23	1492-1894
**Total Atlas Mountains**		42	101	

Altitude was considered at the village and not at the farm level.

### On-farm interviews

In each village, we randomly sampled farmers growing durum wheat, which was the case of all farmers in the Atlas Mountains. A total of 264 farmers was interviewed, 163 from 47 villages in the Pre-Rif, and 101 from 42 villages in the Atlas Mountains. Table 1 shows the distribution of villages and farmers in the different zones, as well as the ranges of elevation.

A semi-structured questionnaire was used. Two main groups of information were collected. First, on the farmer and his farm, questions covered: (i) The age and level of education of the farmer. The level of education was scored in two classes: no education (the farmer never went to school or only to Coranic School) or academic education (from primary school to university, very few farmers being concerned by the later). (ii) Its relationships (yes or no) with the agriculture extension office. (iii) The cultivated area and number of plots of the farm. (iv) The name of all cultivated species, their importance in the farm on a relative scale and the use made of the harvest (percent of self consumption or sale). (v) The livestock: name of the species and their food source. (vi) The level of modernization assessed by the use of mechanization for at least one step of the cultivation cycle, and the use of synthetic fertilizers or pesticides. (vii) The distance to the nearest local market.

Second, the farmer listed the names of all his cultivated varieties. For each variety, he was asked for: (i) its status (traditional versus modern). (ii) The area devoted to its cultivation. (iii) The length of cultivation in the farm, recorded either as the year of first cultivation, or as the statement “ancient” or “very ancient”. (iv) The description of the varieties according to the 16 following traits, referring to morphology and agronomic performance (awn color, spike density, length of spike, seed size, seed color, grain yield, straw yield, type of straw), adaptation (lodging resistance, resistance to diseases), and post-harvest quality (easiness of threshing, of coarse crushing and of grinding, firmness and color of semolina, bread-making quality). Except for qualitative traits like color (seed, awn, semolina) and type of straw, the farmer gave a score on a scale with 3 levels (low, middle or high value of the trait considered). (v) The seed supply: preferred source of seed for planting (production on the farm, purchase from relatives and friends, from local market or from the agricultural office) and reasons underlying this preference. (vi) The selection process: practice of selection (yes or no), stage of selection (seed, plot, spike) and people involved in the process (man only, woman only, farmer and other persons).

### Statistical analyses

A score of modernization was computed for each farmer on a scale of three. A point was assigned when the farmer had access to mechanization, when he used synthetic fertilizers or when he used synthetic pesticides. For each farmer, we computed a measure of the preference for seed self-production, as the proportion of the varieties grown by a farmer for which he declared preferring producing the seed on its farm.

Averages and frequencies were calculated from the survey information to describe the two regions and the different zones. Tests were performed to assess the differentiation of zones within region using the software R [29]: One-way ANOVAs were performed for quantitative variable (function lm), whereas exact tests (function fisher.test) were performed for frequencies.

For each variety, we computed the average geographical distance between farmers growing that variety and the barycentre of all farmers growing that variety: this value was called the geographical dispersion of a variety. Difference of geographical dispersions between status (traditional or modern) was tested by an ANOVA within each region.

Tests were performed to look for differences between two classes of farmers: those growing at least one modern variety and those growing only traditional varieties. ANOVAs were performed for quantitative variables. Fisher exact tests were performed for qualitative variables.

Diversity statistics were computed following Jarvis et al. [11]. Richness is the number of different varieties regardless of their frequencies, and was computed at the farm, zone and region levels. Evenness describes how similar the frequencies of the different variants are, with low evenness indicating dominance by one or a few types [11,30]. Eveness (*Es*) was estimated as:

$$\mathrm{Es}=1-\Sigma \;pi^{2}$$

Where *pi* was the proportion of the cultivated area devoted to the i^th^ variety. It was computed at the farm and zone levels.

Divergence between farms within a zone is a measurement of the proportion of the evenness of the zone displayed among farms. It was estimated as:

$$D=\frac{\mathrm{Es}\left(\mathrm{zone}\right)-\mathrm{Es}\left(\mathrm{farm}\right)}{\mathrm{Es}\left(\mathrm{zone}\right)}$$

Where *Es(farm)* was the average evenness among farms in the zone and *Es(zone)* was the value of the zone evenness.

A multiple correspondence analysis was performed based on the 16 variables used to describe the varieties (see above). The objective of this analysis was to assess the consistency of the description given by different farmers for the same variety, and the differences between different variety names. This analysis was conducted with the software R, using the package *FactoMineR*. Supplementary categorical variables were considered: zone, length of cultivation, selection stage, education level, modernization score and interaction with the agriculture extension office.

## Results

### Farms and farmers: the agro-ecosystems in the study regions

The heads of farms were predominantly male (95% in the Pre-Rif and 98% in the Atlas Mountains), aged between 32 and 77 years (95% interval). On average, 36% of farmers attended at least to primary school. This proportion varied significantly within the Atlas Mountains, with a decrease along the Ziz valley (80% in ZS1, 45% in ZS2 and 7% in ZS3, exact-test *P* = 5.45.10^-6^).

A high proportion of farms, (70%) covered less than 4 ha. In the Pre-Rif, there were no significant differences between zones, the averages within zones being sometimes inflated by a few outliers of more than 50 ha (Table 2). Significant differences for the number of plots, with higher values in ZN4, were observed (*F* = 7.61, *df* = 3, *P* = 8.7.10^-5^). In the Atlas Mountains, significant differences were detected due to the originality of ZS6, which was characterized by larger farms (*F* = 24.42, *df* = 5, *P* = 1.1.10^-15^) with a lower number of plots (*F* = 5, *df* = 5, *P* = 4.10^-4^).

**Table 2:** Average values of some characteristics of the farms in the studied regions

**Zone**	**Farm area (ha) [sd, min-max]**	**Number of plots [sd]**	**Number of crop species [sd]**	**Modernization score [range]**^**a**^	**Relationship with AEO**^**b**^	**Distance to local market (km) [range]**
ZN1	5.01 [3.49, 0.5–20]	6.3 [3.86]	4.51 [1.38]	2.59 [0–3]	39%	3 [1–7]
ZN2	2.91 [2.49, 0.5–6]	5 [2.16]	4.1 [0.99]	3 [3]	10%	5.8 [0–8]
ZN3	5.81 [7.18, 0.7–60]	7.7 [5.21]	4.58 [1.35]	2.43 [0–3]	29%	6.7 [0.3–20]
ZN4	7.63 [11.55, 2–50]	12.4 [6.03]	4.88 [0.72]	2.37 [1–3]	0%	15.3 [14–17]
Pre-Rif	5.56 [6.67, 0.5–60]	7.5 [5.07]	4.56 [1.29]	2.51 [0–3]	28%	6.3 [0.3–20]
ZS1	2.13 [1.5, 0.5–6]	12.5 [5.49]	5.2 [1.01]	2 [1–3]	60%	20.5 [14–30]
ZS2	1.18 [0.65, 0.5–3]	11.2 [3.58]	6.1 [1.34]	1.14 [1,2]	36%	31.6 [0–60]
ZS3	1.84 [1.49, 0.25–6]	13.4 [8.65]	4.33 [1]	0.93 [0–2]	37%	17.6 [0–34.7]
ZS4	1.81 [0.8, 1–3.5]	7.4 [6.37]	4.38 [0.52]	1.87 [1–3]	50%	2.5 [0–5]
ZS5	1.08 [0.38, 0.5–1.5]	12.2 [5.78]	3.33 [1.63]	2 [2]	67%	1.5 [0–3]
ZS6	16.2 [11.28, 2–50]	5.7 [3.31]	6.09 [1.08]	2.26 [1–3]	26%	9.5 [0–16.6]
Atlas Mountains	5 [8.21, 0.25–50]	10.6 [6.52]	5.18 [1.42]	1.57 [0–3]	41%	17.1 [0–60]

*sd*: standard deviation.

^*a*^The score varies from 0 (no use of either mechanization, synthetic fertilizer or synthetic pesticides) to 3 (all these three indicators are used).

^*b*^Percentage of farmers declaring having relationships with the Agriculture Extension Office.

Farmers grew between 1 and 8 different species, with a mode around 4 and 5, and significant variation between zones in the Atlas Mountains (*F* = 13.1, *df* = 5, *P* = 1.1.10^-9^). The main species differed between regions, but also within the Atlas Mountains (Table 3): Zone ZS6 appeared again atypical: two of its main crops (bread wheat and olive tree) were absent from the other zones, while it lacked faba beans, alfalfa and maize, prevalent elsewhere in the Atlas. Durum wheat was cited as important in area in both regions. Quantitatively, it covered a more important relative area in the farms of the Atlas Mountains (around 50%) than in the Pre-Rif (35%), although in smaller areas on average per farm.

**Table 3:** Summary of crops species cultivation and importance of durum wheat in the studied areas

	**Pre-Rif**	**Atlas (ZS1-ZS5)**	**Atlas ZS6**
**Number of different species**	17	14	13
**Most cited species (in addition to durum wheat)**	bread wheat (83%), olive tree (59%), faba bean (81%), barley (91%)	faba bean (31%), alfalfa (51%), maize (31%), potato (30%), apple tree (32%)	bread wheat (31%), olive tree (29%), barley (42%), potato (31%), apple tree (29%)
**Species ranked as the most important in the farm**	Olive tree, durum wheat (83%), bread wheat	durum wheat (91%), alfalfa	durum wheat (86%), bread wheat
**Other non negligible species**	coriander (9%), chick pea (54%)	almond tree (31%), tomato (31%)	plum tree (31%)
**Average durum wheat area per farm (ha)**	1.61	0.75	7.89
**Average proportion of the farm area cultivated with durum wheat**	35%	48%	54%

In brackets is given the rate of self-consumption. All surveyed farms grew durum wheat.

Farming was preferentially oriented to subsistence. Whatever the crop, there was always a portion of self-consumption. In the Pre-Rif, the rates of self-consumption for the main crops were higher than 80%, except for olive trees (60%). Species with less than half of self-consumption were rare. In the Atlas, rates of self-consumption were lower, with values around 50% for alfalfa. For all the other crops, the rates were around 30% (Table 3).

There was a higher level of modernization in the Pre-Rif than in the Atlas Mountains. There were significant differences within the Atlas Mountains (Exact test *P* = 5.10^-4^). However, our data did not allow discriminating between punctual uses of mechanization, synthetic fertilizers and pesticides, from frequent ones. These results thus correspond to an access to these commodities, rather than to a quantitative assessment of their use.

The majority of farmers associated cultivation and rearing. Over the whole 264 farmers, only 5 (from the Pre-Rif region) did not have any livestock. Farmers reared 2.5 different species on average (from 1 to 5), with no difference between regions and zones. The most cited species was cattle. In the Pre-Rif, it was most often associated with donkeys and sheep, and with sheep in the Atlas Mountains. All farmers fed their animals at least partly with straws, except one farmer who used only pasture.

The relationships with the agriculture extension office (AEO) were relatively scarce: only around one third of farmers declared punctual or frequent interaction with it. Differences were significant within the Pre-Rif (Exact test, *P* = 0.0058). Local markets were on average farther in the Atlas than in the Pre-Rif, but with a very high level of variation from farm to farm, and between zones (*F* = 52, *df* = 3, P = 2.2.10^-16^ for the Pre-Rif and *F* = 16.2, *df* = 5, *P* = 1.6.10^-11^ for the Atlas Mountains). In the Pre-Rif, ZN4 was the most isolated, whereas farms in the Ziz valley were on average more isolated, although with a wide variance among farms.

### Traditional and modern varieties of durum wheat

Nineteen different varieties were cited in the Pre-Rif and 14 in the Atlas Mountains, with highly uneven relative frequencies (Table 4). In the Pre-Rif, five varieties were grown on more than 75% of the surveyed area and by 82% of farmers; the same relative area was covered by only 2 varieties in the Atlas Mountains, grown by 50% of farmers. But this observation was mostly due to ZS6, where only two varieties were identified, and where farm areas were much larger than in the other zones: in ZS1 to ZS5, seven varieties were grown in more than 75% of the area and by 87% of farmers. Eight varieties (resp. three) were cited only once or twice in the Pre-Rif (resp. in the Atlas Mountains). Except for the modern variety Karim, no variety name was in common between the two regions.

**Table 4:** Varieties cited in each region

	**Variety**	**Number of citations**	**Status**	**Translation**	**Percentage of farmers**^***a***^	**Percentage of cultivated area**^***b***^	**Geographical distribution**^***c***^
**Pre-Rif**	Elhjaoui	1	traditional	-	0.6	0.2	ZN1
	Guemh	11	traditional	Durum wheat	6.7	2.3	**ZN3**
	*Guemh beldi*	22	traditional	Local durum wheat	13.5	9.4	**ZN1**,**ZN2**,ZN3
	Guemh khel	1	traditional	Black durum wheat	0.6	0.1	ZN3
	*Guemh Lehmar*	35	traditional	Red durum wheat	21.5	18.4	**ZN1**, ZN2
	*Karim*	61	modern	-	37.4	27.7	**ZN1,ZN2,ZN3**
	Krifla beda	13	traditional	White Krifla ^d^	8.0	5.4	**ZN4**
	*Krifla kehla*	35	traditional	Black Krifla ^d^	21.5	11.0	**ZN3,ZN4**
	Lekhel	5	traditional	The black	3.1	1.9	ZN1, **ZN3**
	Massa	3	modern	-	1.8	1.2	**ZN3**
	Marzak	9	modern	-	5.5	5.2	**ZN3**
	Mezrouba	17	traditional	Early	10.4	4.5	**ZN3**
	Ourgh	1	modern	-	0.6	1.5	ZN3
	Pedro	1	modern	-	0.6	0.6	ZN3
	Technique	2	traditional	-	1.2	0.3	ZN1, ZN2
	Twinssia	2	traditional	Comes from Tunisia	1.2	1.1	**ZN3**
	*Zeriâa*	30	traditional	Seeds	18.4	8.7	ZN1, **ZN3**
	Zeriâa twila	1	traditional	long seeds	0.6	0.2	ZN2
	Zeriâi Lkhel	1	traditional	Seeds of the black	0.6	0.1	ZN1
**Atlas Mountains**	Aberyoun	15	traditional	Black awn	14.9	3.9	**ZS2, ZS3**
	Chgira lbida	4	traditional	Small white tree	4.0	2.9	**ZS1**
	Cocorit	7	modern	-	6.9	0.9	**ZS1, ZS2, ZS5**
	*Ifermourgh*	31	traditional	Wing of the grasshopper (six rows)	30.7	59.4	**ZS2, ZS3, ZS6**
	Ilks	2	traditional	Thin spike (four rows)	2.0	0.3	**ZS3**
	Irden taghezaft	6	traditional	Long durum wheat	5.9	1.6	ZS3, **ZS4**, **ZS5**
	Isli	1	modern	-	1.0	0.2	ZS5
	Karim	3	modern	-	3.0	0.5	**ZS2**, ZS5
	Lbida touila	6	traditional	Long white	5.9	1.9	**ZS1**
	Tabekhoucht	3	traditional	The black	3.0	0.7	**ZS2**, ZS4
	Taberyount	6	traditional	Black awn	5.9	2.6	ZS2, **ZS3,** ZS5
	Tamellalt	10	traditional	The white	9.9	2.1	**ZS1, ZS2, ZS5**
	*Toumlilt*	27	traditional	White awn	26.7	22.7	**ZS1, ZS2, ZS4, ZS6**
	Zerbana	1	traditional	Early	1.0	0.2	ZS4

Variety names in italics: The varieties that together covered 75% of the durum wheat area in each region.

‘-’: no translation was found.

^*a*^Percentage of the interviewed farmers having cited the variety. Since farmers could grow more than one variety, the sum of each column is above 100%. ^*b*^Percentage of the total durum wheat area surveyed (i.e. sum of the durum wheat areas over the farmers) where the variety was grown. The values of the column sum to 100%. *c* zones where the variety has been cited. In bold when the variety has been cited at least twice in a given zone. *d* The meaning of Krifla has not been elucidated. It can refer either to a family name or to a region close to Rabat.

In both study regions, the names of traditional varieties referred to morphological (awns and grain color) or agronomic traits (earliness) or to provenance. In some cases, translation was not possible, the name being probably related to a family or an unknown location (Table 4).

The adoption of modern varieties was very different between regions. Among the 19 varieties cited in the Pre-Rif, there were five modern varieties covering 36% of the area allocated to durum wheat and grown by 44% of farmers. In the Atlas Mountains, only 10% of farmers grew the three cited modern varieties, which covered 1.5% of the durum wheat area (Tables 4 and 5).

**Table 5:** Summary statistics on the occurrence and relative importance of modern and traditional varieties

**zone**	**Total number of citations**	**Citations of modern varieties**	**Percentage of farmers growing a modern variety**	**Mean area of plots of traditional varieties (ha)**	**Mean area of plots of modern varieties (ha)**	**Mean percentage of durum wheat farm area devoted to traditional varieties**
ZN1	69	14	27.5	1.31	1.09	87%
ZN2	13	5	50.0	0.4	0.85	59%
ZN3	152	56	61.6	0.75	1.35	61%
ZN4	17	0	0.0	1.15	NA	100%
Pre-Rif	251	75	44.2			
ZS1	17	2	13.3	0.95	0.4	95%
ZS2	28	4	18.2	0.44	0.25	92%
ZS3	28	0	0.0	0.76	NA	100%
ZS4	8	0	0.0	0.75	NA	100%
ZS5	11	5	66.7	0.45	0.4	64%
ZS6	30	0	0.0	6.05	NA	100%
Atlas	122	11	9.9			

Plots with modern varieties were slightly, but not significantly, larger than with traditional varieties, especially in zones where a majority of interviewed farmers grew modern varieties (Table 5). Tests conducted in the Pre-Rif region, and aiming at detecting differences between the characteristics of farmers growing or not modern varieties didn’t yield any significant results, except for the number of cultivated species (*F* = 6.9, *df* = 1, *P* = 0.001, more cultivated species for farmers growing modern varieties).

Varieties were most often cultivated in restricted geographical areas, and sometimes shared between close zones (Table 4). However, it was not rare that representatives from the same variety – modern or traditional – were found far from each other. In the Pre-Rif, the average geographical dispersion (i.e. the average distance of all farms growing a variety to their barycentre) was thus only slightly (and not significantly) higher for modern (21.8 km, 4.6-48.8 range) than for traditional varieties (15.7 km, 1.4-39.8 range), with a high dependency on the number of citations. The average distance between the most distant farmers growing a variety was 65 km, whatever the status. These values were not compared in the Atlas Mountains, due to the rare occurrence of modern varieties.

### Diversity statistics

The richness by zone ranged from 2 to 13 varieties (Table 6). Farmers grew between 1 and 3 different varieties in the Atlas Mountains, and between 1 and 4 in the Pre-Rif. In the Atlas Mountains, 20 farmers out of 101 grew more than one variety. In the Pre-Rif, they were 74 out of 163.

**Table 6:** Estimates of diversity statistics for cultivated varieties of durum wheat

**Region**	**Zone**	**Mean farm richness**	**Zone richness**	**Mean farm eveness**	**Zone eveness**	**Divergence**
**Pre-Rif**	ZN1	1.35	8	0.18	0.61	0.7
	ZN2	1.3	5	0.13	0.59	0.79
	ZN3	1.77	13	0.28	0.8	0.65
	ZN4	1.06	2	0.03	0.33	0.91
**Atlas Mountains**	ZS1	1.13	5	0.06	0.67	0.91
	ZS2	1.27	8	0.13	0.76	0.82
	ZS3	1.04	5	0.02	0.7	0.97
	ZS4	1	4	0	0.68	1
	ZS5	1.83	6	0.36	0.77	0.53
	ZS6	1.3	2	0.14	0.38	0.63

The quite low richness per farm resulted in a low average value of evenness at the farm level. As for richness, evenness was higher when computed at the zone level, which was reflected in the relatively high value of the divergence. This divergence highlighted that the varietal diversity of a zone was maintained by different farms growing different varieties.

### Farmers’ management of their varieties

#### *Seed source and renewal of seed lot and varieties*

In the Pre-Rif, the preference for seed production on the farm was 88% on average among farmers, varying between 79% in ZN3 and 100% in ZN1. In the Atlas Mountains, there was a strong gradient in the Ziz valley from 80% in ZS1 to 16.7% in ZS3, with values around 60% in the ZS4 to ZS6. Modern varieties were largely produced on farm (78.1%), although less often than the traditional ones (Table 7).

**Table 7:** Sources of seed supply according to the region and the status of the variety

	**Status**	**self-production**	**purchase from friends or relatives**	**local market**	**AEO**
*Pre-Rif*	**traditional**	147 (91.3%)	6 (3.7%)	7 (4.3%)	1 (0.6%)
	**modern**	57 (78.1%)	1 (1.4%)	7 (9.6%)	8 (9.6%)
*Atlas Mountains*	**traditional**	60 (53.6%)	8 (7.1%)	42 (37.5%)	1 (0.9%)
	**modern**	2 (22.2%)	0	2 (22.2%)	5 (55.6%)

Given is the number of citations for each source, and the percentage of citations within each status (in brackets).

The major reason cited for on-farm seed production in the Pre-Rif was seed purity and quality. But the same reason was invoked for buying modern varieties to the Agricultural Extension Office. In the Atlas, where seeds were more often bought on the local markets, the reason invoked was seed yield. Farmers producing their seed on their farm do it because they were thus more confident in the identity and quality of the seed.

The adoption of modern varieties was on average more recent than the traditional ones, and their adoption increased in the last years in the Pre-Rif (Table 8). Interestingly, the renewal of varieties at the farm level was overall more rapid in the Pre-Rif than in the Atlas Mountains: a high proportion of the populations grown by the farmers correspond to varieties – whether modern or traditional – that they have introduced in their farm in the last decade. By contrast, less frequent renewal was observed in the Atlas Mountains.

**Table 8:** Distribution of the dates of introduction of the varieties in the farms, according to their status

**Region**	**Status**	**Very ancient**	**Ancient**	**before 1970**	**[1970,1980]**	**[1980,1990]**	**[1990,2000]**	**[2000,2005]**	**[2005,2010]**	**2010**
**Pre-Rif**	**traditional**	109	3	2	1		4	9	17	31
	**modern**	1	1				5	14	29	25
	**Total**	110	4	2	1		9	23	46	56
**Atlas Mountains**	**traditional**			20	16	32	16	5	4	1
	**modern**						3	2	4	2
	**Total**			20	16	32	19	7	8	3

The questionnaires were slightly different in the two regions: in the Pre-Rif, the opportunity was given to farmers to answer by “Very ancient” or “Ancient”, which was not the case in the Atlas Mountains.

#### *Use of the harvest*

On average, a low proportion of the harvest was sold (14%, with no strong difference between regions and zones). Only 40% of the farmers sold part of their production. These farmers declared selling around 40% of it in the Pre-Rif against 25% in the Atlas Mountains. In the Pre-Rif, there was a slightly significant tendency for farmers growing modern varieties to sell a bigger part of their production (21.5% vs. 13.5%, *F* = 4.92, *df* = 1, *P* = 0.028).

#### *Selection*

The selection was not practiced by all farmers. More farmers declared practicing selection in the Atlas Mountains than in the Pre-Rif (but with significant differences between zones in the Pre-Rif, Fisher exact test, *P* = 4.1.10^-6^). Selection stages and actors were different in the two regions, with women more often involved in the Pre-Rif and selecting almost always on seed. By contrast, selection was more a man’s work in the Atlas Mountains, with selection acting both on seed and on plant and spike (Table 9).

**Table 9:** Practice of selection, selection stage and persons involved in the selection in the different zones

**Zone**	**Percentage of farms where selection is practiced**	**Selection stage (%)**^**a**^			**Persons involved in the selection process (%)**^**b**^		
		**seed**	**plot**	**spike/plant**	**man**	**woman**	**farmer (man) and family**
ZN1	71.4	100.0	2.9	5.7	11.4	85.7	2.9
ZN2	85.7	100.0	0.0	0.0	0.0	100.0	0.0
ZN3	30.1	92.0	4.0	28.0	64.0	24.0	12.0
ZN4	37.5	100.0	16.7	33.3	83.3	0.0	16.7
*Nord*	*46.5*	*97.2*	*4.2*	*15.3*	*34.7*	*58.3*	*6.9*
ZS1	100.0	13.3	0.0	86.7	86.7	0.0	13.3
ZS2	95.5	38.1	0.0	61.9	66.7	0.0	33.3
ZS3	81.5	68.2	0.0	31.8	31.8	0.0	68.2
ZS4	75.0	50.0	16.7	33.3	66.7	0.0	33.3
ZS5	100.0	16.7	0.0	83.3	66.7	16.7	16.7
ZS6	78.3	22.2	0.0	77.8	77.8	0.0	22.2
*Sud*	*87.1*	*37.5*	*1.1*	*61.4*	*55.8*	*1.9*	*42.3*

^*a*^The value corresponds to the percentage of farms where selection is practiced at a given stage. Since farmers had the possibility to give multiple answers, the sum of each line is above 100%.

^*b*^The value corresponds to the percentage of the surveyed farms where selection is practiced by a given category of person.

For *a* and *b*, the percentages were computed among the farms where selection is practiced.

Except for 8 farmers (among the 94 that cultivated more than 1 variety), the answers were the same for the different varieties cultivated by a farmer. No significant trend distinguished the selection practices of farmers growing modern varieties or not.

### Farmers’ perceptions of their varieties

**Figure 2:**
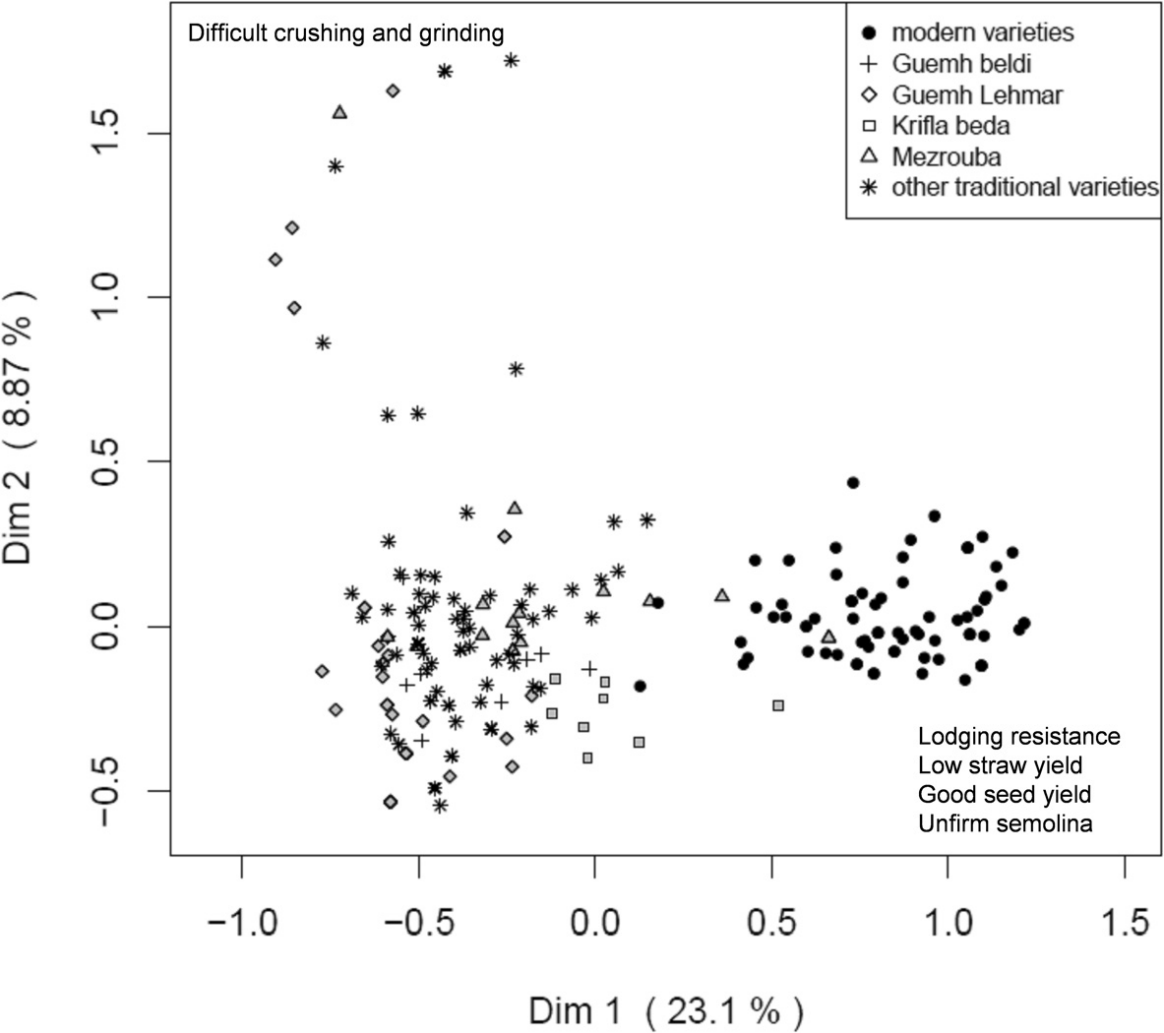
**Multiple Correspondence Analysis of variety descriptors for the Pre-Rif region.** The main traits explaining differentiation along each dimension are indicated.

**Figure 3:**
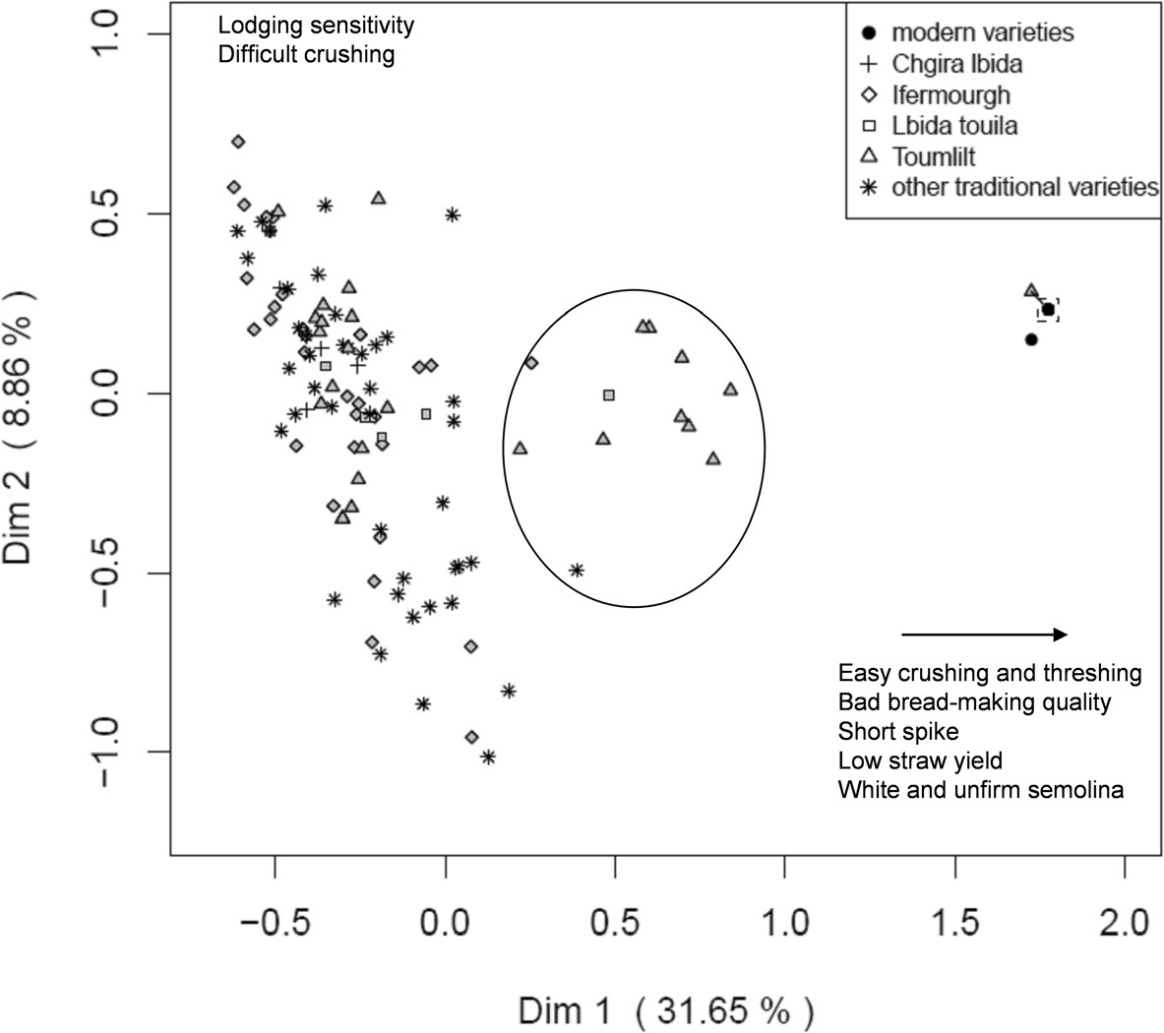
**Multiple Correspondence Analysis of variety descriptors for the Atlas Mountains region.** The square on the right highlights 11 superposed points corresponding to modern varieties. It is also the initial location of the triangle (Toumlilt), which has been slightly shifted to make it visible. The main traits explaining differentiation along each dimension are indicated.

In both regions, the first dimension of the Multiple Correspondence Analysis (MCA) discriminated modern and traditional varieties. Modern varieties were described by rather dense spikes, large and clear seeds and good seed yield, low straw yield and good lodging resistance, easiness of threshing, crushing and grinding, and a rather bad bread-making quality and firmness of semolina (Figures 2 and 3).By contrast, traditional varieties were claimed to have a good straw yield, long spikes, to result in good bread quality, firm and colored semolina. In the Atlas Mountains, threshing and crushing were consistently difficult, whereas they were heterogeneous in the Pre-Rif, with two groups separated on the second dimension (Figure 2).

In both regions, there were a few representatives of traditional varieties that clustered with the modern ones. The modern varieties were more heterogeneous in the Pre-Rif than in the Atlas, but they were also more abundant.In the Atlas Mountains, there was an intermediate cluster that mainly included the traditional variety Toumlilt. This variety showed a wide variation: its representatives occurred all over the graph (Figure 3). The points in intermediate position mainly corresponded to farms in ZS6 and combined traits from the modern and the traditional varieties: good seed yield, average to high straw yield, a difficult threshing, an easy crushing and grinding, good bread-making quality, and rather firm semolina. They thus appeared to combine some better agronomic quality from the modern varieties, with quality traits appreciated by the farmers (straw, and quality of bread and semolina).

In all regions, there was a wide variation within each traditional variety, and no differentiation between representatives carrying different names. In the Atlas Mountains, there was a single trait for which each variety was homogenous: the color of the awn. This was not the case in the Pre-Rif, where only the varieties having black or white in their name (Table 4) were homogenous for awn color.

We detected no evident relationships of this pattern with the supplementary qualitative traits considered in the analysis.

## Discussion

### Contrasted traditional agrosystems, not yet fully characterized

Our data confirmed that in the two regions, our study systems were traditional agrosystems. Namely, farmers practice subsistence agriculture in small plots, combine the cultivation of diverse species with the rearing of animals. In the case of durum wheat, a majority of farmers produce their seeds for sowing and practice selection. This fact had been previously evidenced for other species in the Pre-Rif region barley and faba bean, [15,31]. But these systems are not isolated, in particular from influences of the modernization of agriculture. Farmers have interactions with agricultural extension offices, although these are often limited. Modern commodities are used (synthetic fertilizers, mechanization), especially in the Pre-Rif and some crops are grown for sale.

Beyond this overall diagnostic, contrasted situations occur between and within regions. In addition to the expected differences between the Atlas Mountains and the Pre-Rif – namely, different cultural systems and variable influences from modern agriculture – contrasts are also observed within regions. They can be mainly related to geography and isolation: this is the case of zone ZN4, as well as of the Ziz valley (ZS1 to ZS3) where a gradient is observed for many variables (education, isolation, modernization, altitude). Moreover, a spectrum of practices exists within zones.

This heterogeneity of practices and of agroecologic conditions is a common place, and traditional agrosystems are probably very rarely truly isolated. Many case studies have shown that this heterogeneity is a crucial determinant of the amount, distribution and evolution of agrobiodiversity e.g. [13,32]. One key aspect is missing in our study: the precise ethnic origin of the interviewed farmers and a better understanding of social organization and relationships at the local level. Indeed varieties are social objects and farmers can not be solely considered as individuals, but as members of a society [33]. Getting to a more integrative description of these agrosystems is crucial to understand how their dynamic equilibrium is susceptible to be affected both by climate and economical changes [12,34], in interaction with the social context.

### A diversity of traditional variety names that needs to be complemented by other kinds of information

Traditional varieties of durum wheat are present in the two regions (14 in the Pre-Rif, 11 in the Atlas Mountains); they coexist with modern varieties (5 in the Pre-Rif, 3 in the Atlas Mountains) and are clearly distinguished from them by the farmers (Figures 2 and 3). The majority of farmers grow a single variety, and traditional varieties are mainly cultivated at a local scale, even if names are sometimes found far from each other. Varietal diversity is thus distributed among farms and among zones: As often observed, and as denoted by the level of divergence, the diversity is maintained at the locality and region levels, through different choices made by different farmers [11].

Diversity statistics values are coherent with Jarvis et al. [11]. They appear quite low compared to other species; but not many other references are available for comparison [12,35]. No such estimates exist for wheat in other areas, especially in the area of origin where higher values would be expected. It is then difficult to conclude if our results are a characteristic of durum wheat or of our Moroccan agrosystems. As a comparison, in a study where durum wheat diversity was assessed with microsatellites markers, a lower genetic diversity has been detected in North-West Africa compared to the other parts of the Mediterranean basin [19].

To understand if the diversity of names is a reliable indicator of genetic diversity, one has to unravel to which level names correspond to a unit of management by the farmer “basic diversity units” as explained in [11]. In the Pre-Rif, variability in this correspondence has been evidenced for other species: for barley, one single name is used to refer to a complex of traditional varieties grown by the vast majority of farmers [15], whereas for faba beans, names are much closer to the entities actually distinguished and distinctly managed by the farmers [31]. Here, we recorded more variety names than suggested from a previous study where only two broad categories of durum wheat had been considered namely black and white awn varieties, [27]. However, the perception of farmers (as recorded through a questionnaire) did not allow distinguishing traditional varieties from each other. Since the distinction criteria have been proposed by researchers, they might not reflect the traits that farmers view as important and actually use to describe their varieties [16]. Refined protocols including more consistent information from the farmers (descriptions, confrontations among farmers, demonstration plots) have been applied for other species [11,36]. They appear to be necessary to arrive at correct estimates of traditional variety numbers [16].

Moreover, even if the consistency of variety names is clarified, the extent of variability within and among basic diversity units is crucial to describe the diversity of traditional varieties. For instance, geographic distance, whether associated to environmental variation or not, has been related to genetic variation within traditional varieties of rice [37,38]. The spatial scale of seed exchanges can be quite large as suggested by farmers’ interviews barley in Ethiopia, [32], or genetic data barley in Morocco, [15]. Here, a variety of seed exchange channels coexist; the role of local markets appears especially more important in the Atlas Mountains than in the Pre-Rif (Table 7), and some particular connections have been identified in informal discussions (ZS6 as a seed supplier of ZS3), suggesting that long distance dispersal might be quite important. Reciprocally, names may distinguish varieties that are actually genetically very close e.g. elite varieties of wheat, [17]. Thus, even if variety names are a necessary basis, they are not sufficient to describe genetic diversity. Integrative indicators have been designed e.g. complementing the ones used in Table 6 with parameters of genetic diversity, [17]. Our data thus needs to be complemented by phenotypic and genotypic information.

### Dynamic and open systems: modern varieties adoption, seed renewal and perspective for agrobiodiversity conservation

We evidenced the adoption of modern varieties in villages where traditional varieties are grown. The importance of modern varieties adoption varies between zones: in ZN2 and ZN3 of the Pre-Rif, the majority of interviewed farmers adopted modern varieties, while none did it in ZN4. Adoption is limited in the Atlas Mountains. Isolation from regions where modern agriculture was prevalent (Atlas), from agriculture extension office and/or from communication routes (ZN4, ZS3) might have created fewer opportunities for modern varieties to be adopted. Additionally, in specific environmental and cultural conditions, available modern varieties often don’t cover the farmer’s needs: farmers then get better benefits from their local varieties reviewed in [8].

We couldn’t relate any farm characteristics to the adoption of modern varieties. Their cultivation is not restricted to more modernized farms. In addition, modern varieties were largely maintained on farm (Table 7), and subject to selection. They are integrated into the traditional practices and into the informal seed system.

In addition to being open, the durum wheat system is also dynamic. This is especially true in the Pre-Rif region (Table 8). Adoption of modern varieties is as expected more important in the recent years, but rapid renewal is also evident for traditional varieties. One possible interpretation of the differences between regions might rely on a stronger impact of variable climatic conditions in a rain-fed agriculture: As described for barley, droughts in the Pre-Rif can entail production failures and lacks of seed to sow in the next season [15]. In such situations, farmers have to find another source of seed supply, which might involve introducing a new variety in their farm. In the Pre-Rif, the preference for off-farm sourcing is indeed stronger in bad years (data not shown).

One important question is how this opening and this dynamic might affect the genetic diversity of durum wheat in these agrosystems. The adoption of modern varieties has been shown to be often beneficial for local diversity in a first step (increase of genetic and varietal diversity), as long as they are not predominant in the agrosystems [32,39]. Our interviews are not informative on whether the adoption of modern varieties entails the abandonment of landraces. During the reconnaissance tour, we discarded villages previously known for the cultivation of traditional varieties but where only modern varieties were now grown. This suggests that there is a tendency for the loss of traditional varieties of durum wheat, as has been denoted in other countries in Ethiopia but to the benefit of other crop species, [40]. In a context of increasing climatic pressure, production failures and greater prevalence of modern agriculture could accelerate landrace replacement.

As for maize [41], the integration of the modern varieties in the traditional systems might induce their “creolization” (e.g. intermixing and recombination with traditional varieties and creation of new genetic combinations, although wheat is a selfing species). Reciprocally, mixing can also entail introgression of modern material into the local varieties [42], which can make the impact of modern varieties more important than what the interviews suggest.

Reciprocally, farmers’ preferences for landraces, as evidenced by the qualities they recognize to them (e.g. quality of use, straw yield, Figures 2 and 3), could slow down their disappearance. Some farmers keep at least small plots for traditional varieties even when they “officially” grow modern varieties only (informal discussions). This phenomena has been evidenced for maize in China, even if it doesn’t fully protect landraces from abandonment the reduction of the overall area of landrace cultivation and their more limited presence in informal seed exchange put them at risk anyway, [14]. We now need a better assessment of the reasons of adoption of modern varieties and on their fate once they are incorporated into the traditional systems.

## Conclusions

The surveyed traditional agrosystems constitute open, dynamic and heterogeneous entities; the Pre-rif area is presently more open to influences from outside, in the form of modern facilities (outputs, mechanization) and improved varieties, than the Oases of the Atlas Mountains. These agrosystems still host traditional varieties of durum wheat, although in a relatively moderate number, and their heterogeneity is a factor of maintenance of this varietal diversity, through the different choices made by different farmers. Although the farmers clearly perceive agronomic and quality differences between modern and traditional varieties, they apply to them the same practices of seed management and breeding. To get an integrative view of this varietal diversity and its dynamic, more knowledge is now required on the actual differences between traditional varieties (basis of folk taxonomy, phenotypic and genotypic characterization) and on the factors favouring the advent of modernization and governing the seed exchange practices, including the social structures which are missing here.

Rapid seed renewal at the farm level, increased crop failures and integration of modern varieties into the traditional practices could accelerate the loss of original durum wheat varietal and genetic diversity in the Pre-Rif region. Simultaneously, local preferences for the particular quality traits of traditional varieties (bread and semolina), could favour their maintenance, but potentially only in small plots for self-consumption.

The persistence of traditional crop diversity in a context of agriculture modernization and intensification has become a major sustainability issue and the conditions of coexistence have started to be addressed in some regions [12]. However, it is not only a matter of protecting these agrosystems against modernity. The issue of importance is to valorise the crop diversity present in traditional agrosystems, so that in addition to maintain agrobiodiversity, it benefits to the farmers. This valorisation needs to be anchored in a better knowledge on the factors affecting the evolution of biodiversity: In our case, it implies for instance understanding factors underlying the patterns of distribution of the varieties along the Ziz valley social context, local adaptation to environment and local preferences, [6,8,33]. Including the contribution of modern varieties and scientific knowledge can then to be done in coherence with what already exists [3].

## Competing interests

The authors declare that they have no competing interest.

## Authors’ contributions

LC conducted the data collection, carried out the analyses and the interpretation of the results and drafted the manuscript. AS conducted the data collection and contributed to the interpretation of the results. MA contributed to the design and coordination of the study, to data analysis and interpretation of the results. LB and AB participated in the conception, design and coordination of the study, and contributed to the interpretation of the results. PR participated in the conception, design and coordination of the study, contributed to data analysis and interpretation of the results and helped draft the manuscript. MHM participated in the conception, design and coordination of the study, carried out the data analysis and interpretation of the results and drafted the manuscript. All authors read and approved the final manuscript.

## Authors’ information

LC and AS are MSc at the Institut Agronomique et Vétérinaire Hassan II (Rabat, Morocco); this study forms part of their PhD project. MA, LB and AB are PhD, teachers and researchers at the Institut Agronomique et Vétérinaire Hassan II (Rabat, Morocco). PR and MHM are PhD, researchers at the Institut National de la Recherche Agronomique (UMR Amélioration Génétique et Adaptation des Plantes méditerranéennes & tropicales, Montpellier, France).
